# Hopelessness in patients with schizophrenia

**DOI:** 10.1192/j.eurpsy.2021.1419

**Published:** 2021-08-13

**Authors:** C. Laginhas, D. Jeremias, D. Rodrigues, R. Medinas, N. Moura, C. Pereira

**Affiliations:** Psychiatry Department, Ocidental Lisbon Hospital Center, Lisboa, Portugal

**Keywords:** Hopelessness, Suicide, schizophrénia

## Abstract

**Introduction:**

Individuals with schizophrenia have a shortened average life expectancy, with a lifetime risk of suicide around 5%.

**Objectives:**

Here we present a case of a patient diagnosed with schizophrenia who developed depressive symptoms with suicidal ideation, reactive to psychotic symptoms. Considering this specific case, the factors that contribute to the increased risk of suicide in these patients are reviewed.

**Methods:**

Relevant clinical information was extracted from the patient’s clinical process. In addition, we searched Pubmed^R^ database with the terms “Schizophrenia”, “Hopelessness” and “Suicide”.

**Results:**

A 40-year-old male patient, single and unemployed presents a progressive psychotic condition, with 20 years of evolution, with an impact on social and work behaviour. As a background he has a history of depressive episodes with suicidal ideation at the age of 36, following psychotic symptoms. This is a patient with preserved cognitive functioning combined with a high level of education, who understands the impact of his reality on his functioning. In this context, he develops feelings of hopelessness, that are the risk factor for suicide, most consistently reported in patients with schizophrenia.

**Conclusions:**

This case assesses a patient with schizophrenia who has several factors, that contribute to an increased risk of suicide, focusing on hopelessness. In the future, it may be interesting to study in more detail the individual weight of each factor, so that it is possible to accurately predict the individual risk of each patient and, consequently, it is possible to implement preventive strategies.

